# Nonlinear and inequality-related heterogeneity in green cooling across Chinese urban agglomerations: a SHAP-based random forest analysis

**DOI:** 10.3389/fpubh.2026.1913281

**Published:** 2026-08-06

**Authors:** Zimo Zhang, Zhijie Yang, Luoman Ouyang

**Affiliations:** 1 College of Landscape Architecture, Nanjing Forestry University, Nanjing, China; 2 School of Architecture, Southeast University, Nanjing, China; 3 State Key Laboratory of Resources and Environment Information System, Institute of Geographical Sciences & Natural Resources Research, Chinese Academy of Sciences, Beijing, China; 4 Sichuan Institute of Land and Spatial Planning, Chengdu, China

**Keywords:** Gini coefficient, green cover, green equity, nonlinear effects, urban thermal environment

## Abstract

Land surface temperature (LST) is increasing with rapid urbanization and climate warming. However, the relative roles of green quantity, vegetation condition, and inequality in local green resource availability in shaping the urban thermal environment remain insufficiently understood across regions. This study examined built-up areas in five major Chinese urban agglomerations. A Random Forest model combined with SHAP analysis was used to quantify the relative importance of green-related, natural background, built-environment, and socio-economic variables and to identify their nonlinear associations with LST. Green-related variables contributed consistently across the five urban agglomerations, although their contribution structures differed markedly among regions. NDVI generally showed stronger explanatory power than green cover. The weighted Gini coefficient of local green resource availability exhibited a strongly nonlinear and region-dependent association with LST. Cross-regional comparisons indicated greater sensitivity to changes in the weighted Gini coefficient in the Yangtze River Delta and weaker responses in the Chengdu–Chongqing region. SHAP results further indicated that built-environment morphology was also relevant alongside green-related variables. Built-up density consistently showed positive SHAP values. These findings provide interpretable cross-regional evidence for heat-mitigation planning. Effective strategies should prioritize vegetation quality, the spatial continuity of green resources, and a more balanced distribution of green space rather than focusing solely on the total quantity of green space.

## 1 Introduction

As an ecological consequences of urbanization under global warming, cities and their dense populations are increasingly affected by urban climate issues such as the urban heat island effect (1, 2), which has been shown to impair residents' thermal health and comfort (3, 4), and increase the risk of heat-related diseases and deaths (5). Recent evidence has further revealed pronounced spatiotemporal disparities in exposure to heat-related illness risk and highlighted the role of socio-spatial drivers in shaping urban heat vulnerability (6). Consequently, the impacts and risks associated with climate change are posing significant challenges to the rapid development of cities (7).

Land surface temperature (LST) is an important variable in urban climate research (8) and has been increasingly used to investigate urban thermal environments and urban climate change (9, 10). In the context of rapid urban growth, urbanization and urban land-use change directly influences on LST dynamics (11, 12). In impervious areas with high densities of building and road cover, surface materials typically exhibit higher solar radiation absorption, heat capacity, and thermal conductivity than vegetated and permeable surfaces that support evapotranspiration (13). Current research on LST has focused on multiple factors, including urban topography and building morphology (14–16), and their relationships with LST have frequently been shown to be nonlinear (17). Accordingly, an increasing number of studies have used statistical and machine learning approaches to model and predict LST. Some studies have evaluated the predictive performance of four machine learning algorithms, including K-nearest neighbor regression and neural networks (NN), in modeling LST (18–22). While the value of machine learning approaches in urban thermal studies is widely acknowledged, recent studies have increasingly incorporated interpretable or explainable machine learning techniques to examine how urban morphology and environmental factors are associated with LST. For example, machine learning models enhanced by Explainable Artificial Intelligence (XAI) have been used to reveal the nonlinear contributions of greenness, and built-environment indicators to LST variation in specific cities or local contexts (23, 24). However, most of this evidence remains case-specific, and relatively little is known about whether the relative importance and nonlinear threshold ranges of these predictors are consistent across different urban agglomerations (UAs). In particular, existing interpretable analyses have more often emphasized conventional greenness or morphology indicators, such as vegetation condition, green cover, and built-environment characteristics, whereas the potential role of inequality in the spatial distribution of green resources has received less explicit attention in urban thermal studies.

Among conventional greenness indicators, vegetation indices such as NDVI (Normalized Difference Vegetation Index) and measures of urban green space (UGS) have been consistently reported to correlate with LST (25, 26). Ecosystem services provided by urban green space not only underpin urban ecological integrity (27) but also contribute to thermal regulation. Vegetation can provide shade and absorb solar radiation, thereby reducing summer LST (28–30). In addition, vegetation can attenuate wind, which may increase winter LST (31). Beyond green quantity and vegetation condition, a substantial body of research has highlighted social inequality in the distribution of and access to urban green space (27, 32, 33). The spatial allocation of green space and the benefits it confers are often shaped by socio-economic factors such as income and land values (34, 35). At broader administrative scales, inequality in green space exposure has also been linked to variations in economic development, urbanization processes, and infrastructure investment, suggesting that green equity is shaped by multiple dimensions of urban development (36, 37). For example, higher income and higher educational attainment have been associated with greater availability of nearby green space (38). From a spatial perspective, green cover often varies along the urban gradient from city centers to peripheral areas (39), and these patterns differ across countries with varying levels of urbanization and population density (40).

Although a growing body of work has examined urban green space and its cooling-related benefits, research in China that explicitly links green space inequality to climate-related outcomes remains relatively limited. To date, most Chinese studies on green space have focused on evaluating accessibility, optimizing spatial configurations, and characterizing spatiotemporal change (41–45). Research on green space inequality has primarily addressed the spatial patterns of inequity within cities and has largely been conducted as single-city studies. For example, You (46) found that, in Shenzhen, the quality index describing the spatial distribution of park green space was negatively associated with income, the proportion of the floating population, and housing-related variables, but showed no statistically significant associations with occupation or educational attainment. Zhang et al. (47) investigated the relationship between green space exposure inequality and wet-bulb globe temperature in Wuhan by utilizing wet-bulb globe temperature to characterize thermal comfort and assessing green space exposure inequality with Lorenz curves and the Gini index to reveal their spatiotemporal correlation. Wang et al. (48) evaluated the spatial accessibility of both heat risks and cooling benefits in Fuzhou. Wu and Kim (49) examined intercity green space inequality across China and concluded that access to green space was more uneven in cities in central and southern China, along the southern coast, and in the northeast, whereas cities in western China exhibited more equitable access. Taken together, and considered alongside prior research on the cooling effects of green space, the existing evidence suggests that studies have often addressed either green space equity, with an emphasis on intracity patterns (50, 51), or temporal trends in cooling effects alone (52). Studies investigating the relationship between the thermal environment and green space factors have tended to focus on either a single vegetation parameter (53), urban public service land that includes green spaces (54), or the mechanisms underlying vegetation cooling effects after these effects have been quantified (55). The cooling benefits generated by vegetation are not evenly distributed across space. Differences in the amount, accessibility, and spatial configuration of green space can shape the extent to which residents in different areas are exposed to or protected from surface heat. This relationship is also likely to vary with climatic background and urban development intensity, suggesting potentially nonlinear and context-dependent associations. Such linkages remain insufficiently explained in the existing literature. In addition, comparative work across cities or UAs at broader spatial scales remains limited.

Overall, the existing literature suggests several unresolved issues. Although machine learning methods have been increasingly introduced into urban thermal studies, much of the available evidence remains case-specific, and cross-regional comparisons across UAs are still limited. In addition, previous studies have more often examined conventional greenness indicators, such as NDVI and green cover, rather than the equity dimension of green resources, while research on green space inequality has rarely been integrated directly into analyses of LST and its nonlinear drivers. As a result, it remains unclear how conventional greenness indicators and green resource inequality are jointly related to urban thermal patterns and whether these relationships are consistent or heterogeneous under different climatic and urban development contexts. Addressing these gaps requires a unified and interpretable framework that can compare these relationships across multiple UAs.

This study employs a machine learning framework that integrates Random Forest (RF) with Shapley Additive Explanations (SHAP) and focuses on the built-up areas of five major UAs in China that are broadly representative in terms of geographic location and climatic characteristics. From the perspective of green-related cooling and inequality under regional climatic heterogeneity, we estimate the nonlinear relationships between LST and a set of explanatory variables, including green-related and natural environmental variables as well as socio-economic and urban built-environment variables. On this basis, we aim to (1) quantify the nonlinear relationships of NDVI, green cover, and inequality in the local distribution of green resources, as measured by a weighted Gini coefficient, with LST in built-up areas; (2) compare the relative cooling-associated contributions of these variables and their potential interaction patterns under different levels of green cover and spatial configuration; (3) compare these relationships and cooling contributions across different UAs, assessing the extent to which the identified nonlinearities and relative effects are consistent or heterogeneous among regions. The results are expected to provide actionable quantitative evidence and planning guidance for community-level thermal environment optimization and the refined allocation of urban green space.

## 2 Materials and methods

### 2.1 Study area

**Figure 1:**
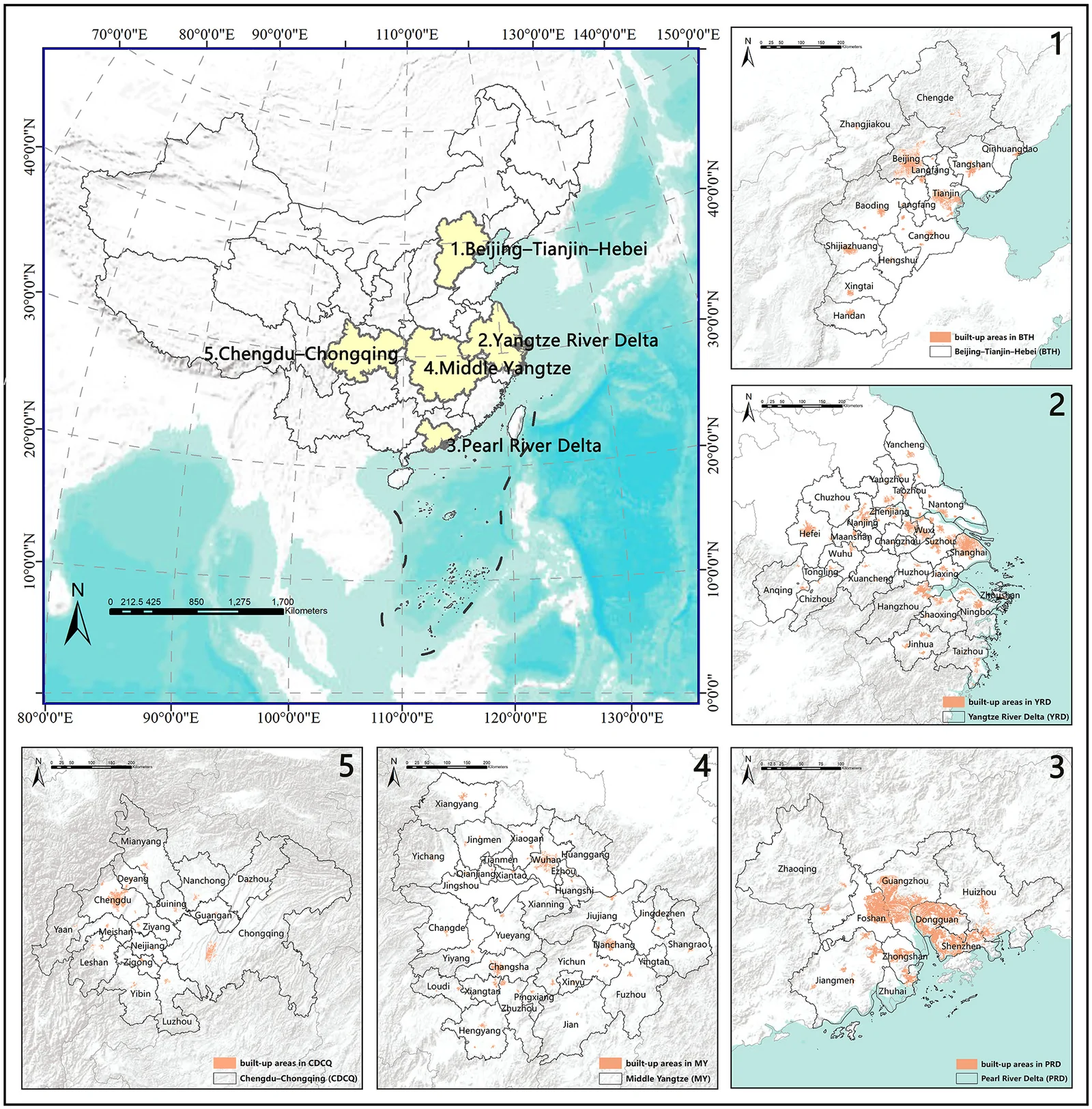
Study area.

Given China's economic growth and the current stage of UA development, this study selected five relatively mature and UAs that provide a comparative sample across different geographic and climatic contexts in China (56), the Beijing-Tianjin-Hebei (BTH), Yangtze River Delta (YRD), Middle-Yangtze (MY), Chengdu-Chongqing (CDCQ), and Pearl River Delta (PRD) UAs (Figure 1). These urban agglomerations are distributed across northern, eastern, southern, and central China and have become important regions in national economic development (57). For these highly urbanized and densely populated UAs, sustainable development has become a central priority (58), requiring continued efforts to advance coordinated social and environmental development.

This study focuses on built-up areas within each UA. Built-up areas refer to highly urbanized spaces that typically encompass urban cores and parts of the urban-rural fringe (43, 59, 60) We used the built-up area dataset produced by Sun et al. (61) from impervious surface extraction, which was generated using Sentinel 1 and Sentinel 2 data and aligned with the United Nations definition of built-up areas, to delineate the analysis boundary.

### 2.2 Data collection

#### 2.2.1 Natural environmental variables

NDVI is widely used to capture reflectance characteristics associated with chlorophyll activity in vegetation (62). It is also a common indicator of vegetation condition and urban greenness (63). In this study, NDVI data were obtained from the Remote Sensing Team for Land Use and Global Change at the Institute of Geographic Sciences and Natural Resources Research, Chinese Academy of Science (64). Landsat observations acquired during the growing season capture vegetation cover, growth conditions, and vegetation vigor. Accordingly, we used an NDVI threshold approach to distinguish green from non-green areas and to delineate the extent of urban green cover (44, 65).

Relative humidity (RH) data were derived from the monthly meteorological gridded dataset provided by the National Cryosphere Desert Data Center, with a spatial resolution of 1 km. The digital elevation model (DEM) was obtained from a dataset developed by the National Aeronautics and Space Administration. LST data for thermal environment analysis were obtained from the NASA Earthdata MOD11A2 product. The original product provides 8-day composite LST data at a spatial resolution of 1 km.

#### 2.2.2 Socio-economic and urban built-environment variables

Population density data used in this study were obtained from the WorldPop platform and consist of annual gridded total population estimates at 100 m resolution that were adjusted using United Nations national population estimates. Built-up density within built-up areas was derived from the JRC Global Built-Up Surface dataset for 1975–2030, which has a spatial resolution of 100 m and a temporal resolution of 5 years. Road density was computed from road transport vector datasets by aggregating road information to a 1-km grid.

To represent building-induced obstruction, we used the Sky View Factor (SVF). SVF is defined as the solid angle Ω of the visible sky from a given observation point, normalized by the solid angle of the upper hemisphere 2π. In practice, SVF can be approximated by estimating the vertical elevation angle of the horizon or surrounding obstructions in a set of azimuth directions. Under this approximation, SVF at location *p* can be calculated as Equation 1 (66):


$$\begin{array}{l} SVF\left(p\right)=1-\frac{1}{n}\overset{n}{\underset{i=1}{\sum }}\sin\gamma_{i} \end{array}$$ (1)

where *n* is the number of directions used to estimate horizon elevation angles, and γ_*i*_ is the vertical horizon elevation angle in direction *i*. SVF ranges from 0 to 1. Values closer to 1 indicate a larger proportion of visible sky in the upper hemisphere, whereas values closer to 0 indicate stronger obstruction.

We calculated SVF using a 10 m resolution building height raster and aggregated the results to a 1 km × 1 km fishnet grid. For each 1 km grid cell, the cell center was used as the observation location, and surrounding building height pixels within a 1 km search radius were scanned in multiple directions to identify the maximum obstruction height in each direction. These obstruction heights were then converted to the corresponding elevation angles γ_*i*_, and SVF was calculated using the above equation. The computed SVF value was assigned to the corresponding 1 km grid cell. At this spatial resolution, SVF should be interpreted as a neighborhood-scale indicator of overall urban openness and building-induced obstruction, rather than as a street-canyon or pedestrian-level microclimate metric. The data sources, spatial resolutions of all variables used in this study are summarized in Table 1.

**Table 1:** Natural environmental, socio-economic, and urban built-environment variables.

Variable	Description	Source	Unit	Resolution
(1) Natural environmental				
NDVI	Normalized Difference Vegetation Index	NDVI computed from cloud-/shadow-masked Landsat 5/7/8/9 via Google Earth Engine https://earthengine.google.com/	/	30 m
Green cover	NDVI-derived green cover	Derived from NDVI	/	30 m
RH	Monthly gridded relative humidity	National Cryosphere Desert Data Center, monthly meteorological gridded dataset: http://www.ncdc.ac.cn/	/	1 km
DEM	Digital Elevation Model	NASA Earthdata (reprocessed SRTM with ASTER GDEM, ICESat/GLAS, PRISM, etc.): https://www.earthdata.nasa.gov	m	250 m
LST	Land Surface Temperature	NASA Earthdata MOD11A2: https://www.earthdata.nasa.gov	°C	1 km
(2) Socio-economic and urban built-environment				
Pop	Population density	WorldPop annual population grid https://hub.worldpop.org/	/	100 m
BD	Built-up density to indicate urbanization intensity	JRC/GHSL GHS_BUILT_S https://ghsl.jrc.ec.europa.eu/	/	100 m
RD	Road density	Derived from road vector datasets	km/km^2^	100 m
SVF	Sky View Factor	$SVF=1-\frac{1}{n}\sum_{i=1}^{n}\text{sin}\gamma_{i}.$	/	1 km

### 2.3 Quantifying inequality in the spatial distribution of local green resources

All variables were measured on a 1-km gridded basis. Following prior studies, we used NDVI-derived vegetated area within each built-up grid cell as an indicator of local green resources availability and defined available green cover based on NDVI (49, 67).

We then quantified inequality in the spatial distribution of these local green resources. For each 1 km × 1 km grid cell center, we constructed a surrounding neighborhood and applied a Gaussian distance decay weight to all grid cells within that neighborhood as a function of their distance to the focal cell. We multiplied the population weight by the Gaussian distance weight to obtain an effective weight. The neighborhood cells were subsequently ranked by the amount of NDVI-derived vegetated area, and the area under the weighted Lorenz curve was computed to derive a weighted Gini coefficient for the focal grid cell (68, 69). Lower values indicate a more even distribution of nearby green resources across the population-weighted neighborhood, whereas higher values indicate greater local concentration. In this study, the weighted Gini coefficient is interpreted as a proxy for inequality in the local green resource availability and, in the thermal context, may also reflect the concentration, continuity, or fragmentation of green-space configuration rather than normative equity in a strict accessibility sense.

The process is as follows:

(1) Gaussian distance weight (Equation 2)


$$\begin{array}{l} k_{gi}=\exp\left(-\frac{d_{gi}^{2}}{2h^{2}}\right),i\in N\left(g\right) \end{array}$$ (2)

For each focal grid cell *g*, a neighborhood *N*(*g*) is constructed. Each neighboring cell *i* is assigned a Gaussian distance decay weight based on its distance to the focal cell, *d*_*gi*_.

(2) Effective population weight (Equation 3)


$$\begin{array}{l} p_{gi}^{eff}=p_{i}k_{gi} \end{array}$$ (3)

The effective weight combines population size and spatial proximity. It is used to represent the relative importance of each neighboring cell in the local inequality calculation.

(3) Weighted Gini coefficient (Equation 4)


$$\begin{array}{l} G_{g}=1-\overset{n_{g}}{\underset{i=1}{\sum }}\frac{p_{g\left(i\right)}^{eff}}{P_{g}}\left(B_{g,i-1}+B_{g,i}\right) \end{array}$$ (4)

We first compute cumulative shares and then estimate the area under the weighted Lorenz curve using a discrete trapezoidal approximation, yielding the weighted Gini coefficient *G*_*g*_ for the focal grid cell *g*.

Here, *P*_*g*_ denotes the sum of effective weights within *N*(*g*). *B*_*g, i*_ denotes the cumulative weighted share of NDVI-derived vegetated area up to rank *i*, where the neighborhood cells are sorted in ascending order by *a*_*i*_, the NDVI-derived vegetated area in cell *i*, and *n*_*g*_ is the number of cells in *N*(*g*).

### 2.4 Methodology

#### 2.4.1 Application of RF

**Figure 2:**
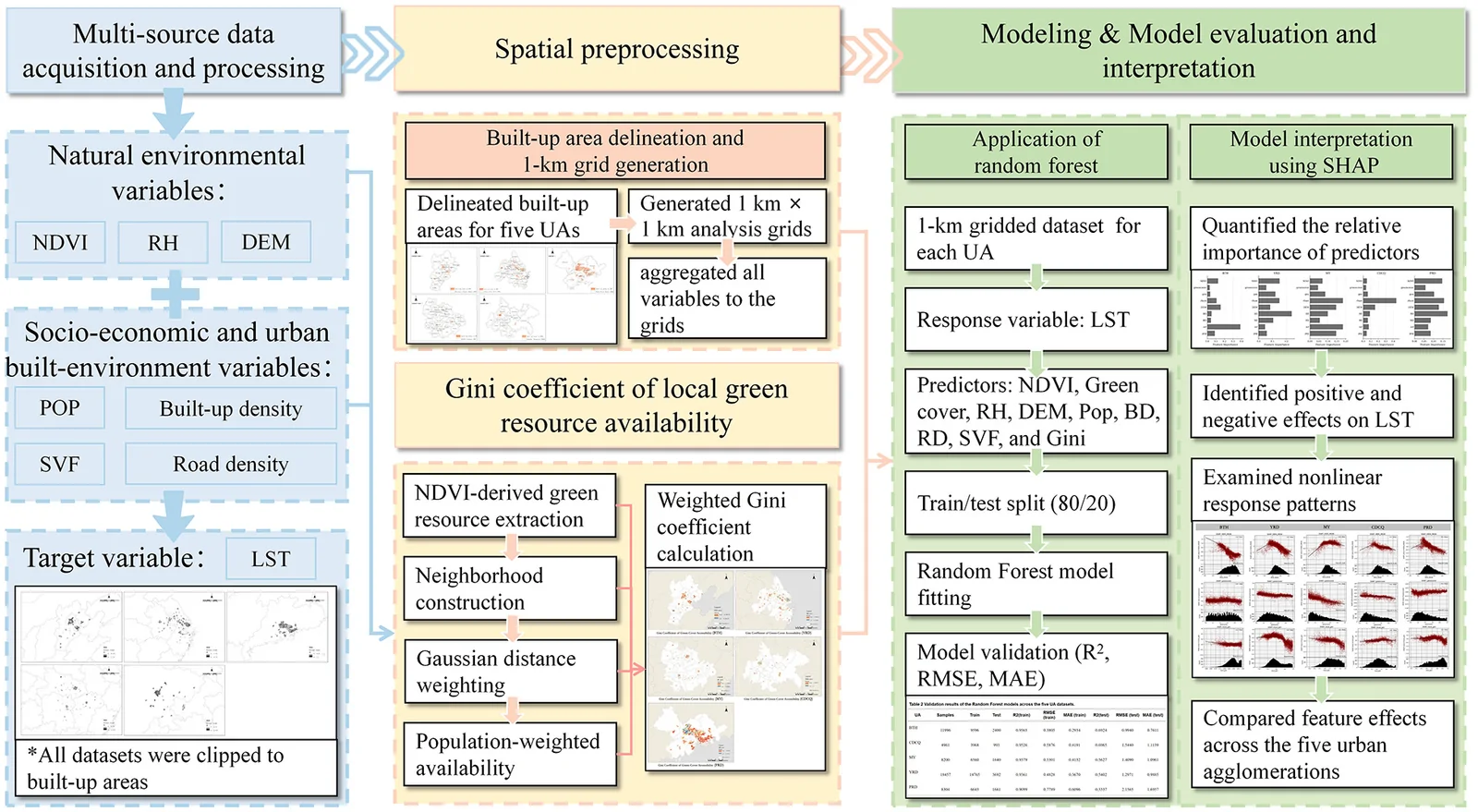
Flowchart of the study.

The overall research workflow, including data collection, spatial processing, and model interpretation is illustrated in Figure 2. RF is an ensemble regression approach based on decision trees. It constructs multiple regression trees using bootstrap resampling and random feature selection at each split, and then aggregates their outputs to improve generalization performance and robustness (70). Given the potential nonlinearities and interactions among LST, urban development, vegetation, and meteorological variables, we employed an RF regression model to predict LST. The random forest prediction function is defined as the average output of *T* regression trees (Equation 5):


$$\begin{array}{l} ŷ=f\left(\text{x}\right)=\frac{1}{T}\overset{T}{\underset{t=1}{\sum }}h_{t}\left(\text{x}\right) \end{array}$$ (5)

where *h*_*t*_(·) denotes the *t*-th regression tree and *T* is the number of trees. The feature vector included *p* = 9 explanatory variables, namely NDVI, green cover, RH, DEM, population density (Pop), built-up density (BD), road density (RD), sky view factor (SVF), and the weighted Gini coefficient of local green resource availability.

#### 2.4.2 Model interpretation using SHAP

To model the relationship between urban predictors and LST, we trained a RF regression model separately for each UA. Each model included 9 predictor variables. Samples were randomly divided into training and test sets at a ratio of 80% and 20%, respectively, using a fixed random seed of 42. The models were trained with 200 trees, with the minimum number of samples in each leaf node set to 1, while the remaining hyperparameters followed the default settings unless otherwise specified. Model performance was evaluated using the coefficient of determination (*R*^2^), root mean square error (RMSE), and mean absolute error (MAE) on both the training and test sets.

To further evaluate model generalization under spatially independent conditions, we conducted a regular spatial block cross-validation. For each UA, samples were grouped into two-dimensional spatial blocks based on their X and Y coordinates. Specifically, the X and Y dimensions were each divided into three rank-based quantile bins, forming a 3 × 3 regular grid. The resulting grid cells were then assigned to five spatial folds in a deterministic round-robin manner. In each iteration, one spatial fold was used as the validation set, and the remaining folds were used for model training. Coordinates were used only for spatial grouping and were not included as predictors. Model performance under spatial cross-validation was evaluated using the same metrics, namely the coefficient of determination (R^2^), root mean square error (RMSE), and mean absolute error (MAE).

To identify and quantify the contribution of each variable to model-predicted LST and its direction, we interpreted the model using SHAP. SHAP values for the tree-based model were computed with TreeExplainer to quantify the contribution of each feature to individual predictions.

Formally, SHAP approximates the sample level output of the original model with an interpretable additive function (Equation 6):


$$\begin{array}{l} g\left({\text{z}}^{\prime }\right)=\phi_{0}+\overset{M}{\underset{j=1}{\sum }}\phi_{j}z_{j}^{\prime } \end{array}$$ (6)

where ϕ_0_ is the baseline term, which typically corresponds to the overall mean predicted value, *M* is the number of features, ϕ_*j*_ is the attribution value for feature *j* (the Shapley value), and $z_{j}^{\prime }\in \{0,1\}$ indicates whether a feature is included in a coalition. For a given sample *i*, this can be expressed as (Equation 7):


$$\begin{array}{l} f\left({\text{x}}_{i}\right)=\phi_{0}+\overset{p}{\underset{j=1}{\sum }}\phi_{ij} \end{array}$$ (7)

where ϕ_*ij*_ > 0 indicates that feature *j* increases the predicted LST above the baseline, and ϕ_*ij*_ < 0 indicates that it decreases the predicted LST.

For each feature *x*_*j*_, we characterized the relationship between feature values and SHAP magnitudes by constructing SHAP dependence plots. Apparent turning ranges were identified from the smoothed SHAP dependence curves and marked in the figures using vertical shaded bands. These ranges are intended to guide interpretation of nonlinear model-associated patterns. Specifically, we plotted the feature value *x*_*ij*_ on the horizontal axis and the SHAP value ϕ_*ij*_ on the vertical axis, and overlaid a nonparametric smoothed curve to show the overall trend. We also added a histogram of the feature distribution at the bottom of each plot to indicate sample density and reduce the risk of overinterpreting trends in sparsely populated value ranges. Based on these interpretive outputs, we further exported the SHAP value matrix, scatter-based regression summaries, and marginal histograms to support cross-variable and cross-UA comparisons of relative contributions and model-identified association patterns.

## 3 Results

### 3.1 Spatial distribution of the weighted Gini coefficient of green resource availability across UAs

**Figure 3:**
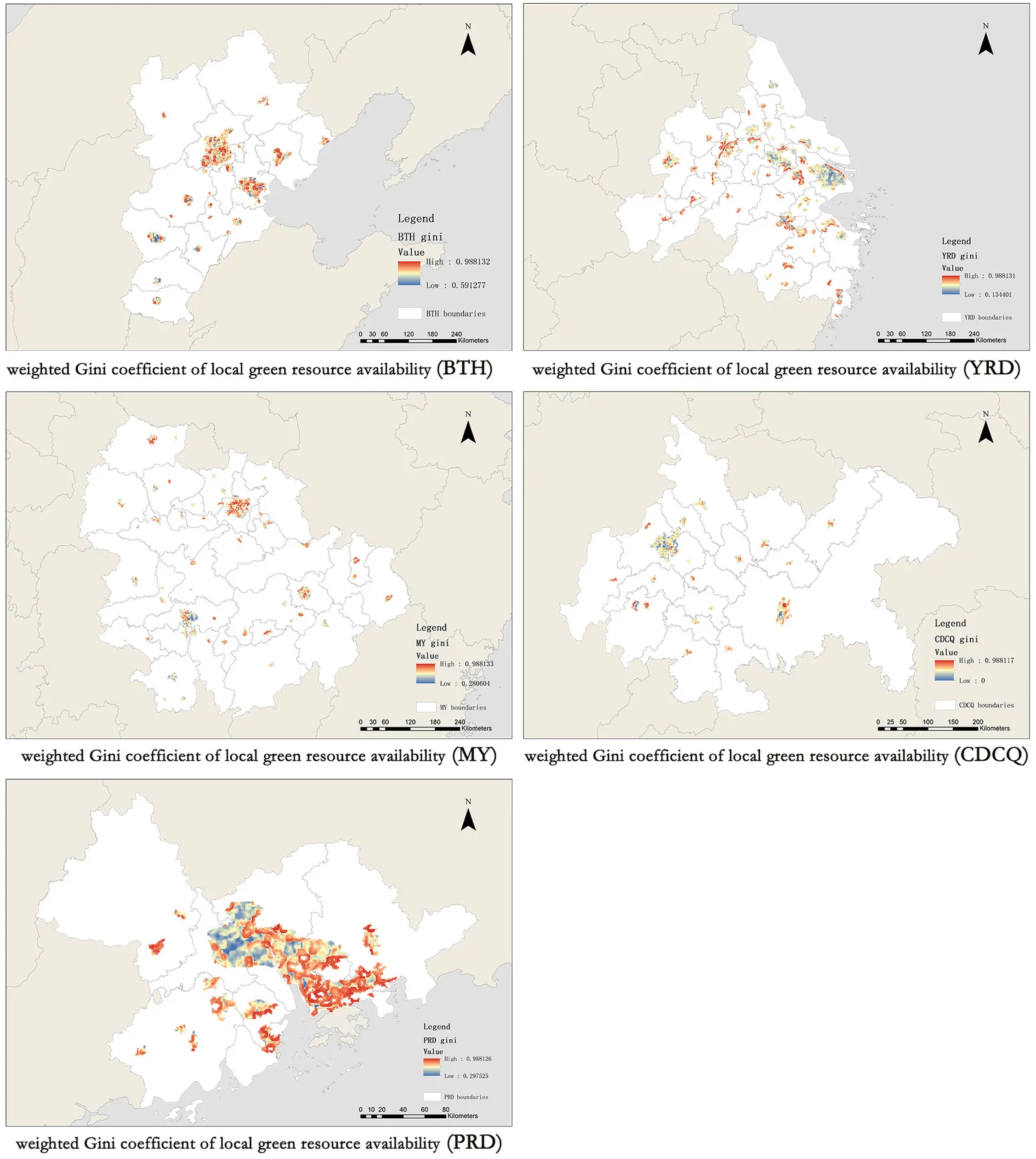
Spatial distribution of the weighted Gini coefficient of green resource availability across the five UAs.

Figure 3 illustrates the spatial distribution of the Gini coefficient of local green resource availability across the built-up areas of the five UAs. Overall, the Gini coefficients exhibit a pattern characterized by localized clustering of high values and a more scattered distribution of low values. Areas with high Gini coefficients tend to appear as patch-like or contiguous clusters, whereas low-value areas are relatively dispersed and form continuous belt-like or areal patterns in some locations. Across UAs, YRD and CDCQ show comparatively lower overall Gini coefficients, with some subareas showing a distinct distribution of values well below 0.5. In contrast, the other three UAs display Gini coefficients predominantly above 0.5, with BTH showing the highest overall levels.

Within each UA, comparatively developed built-up areas, such as those in Shanghai, Chengdu, Guangzhou, and Foshan, show a denser higher concentration of low Gini coefficients than less developed areas. Meanwhile, the relatively extreme high and low values also tend to be concentrated in built-up areas of larger cities. In contrast, in less developed cities, Gini coefficients within built-up areas are often above 0.6, and high value areas tend to show an outward gradient toward the urban periphery.

### 3.2 Model performance and validation results

Table 2 summarizes the performance of the RF models under the conventional random train-test split across the five UA datasets. Overall, the models showed high goodness of fit on the training sets, whereas performance on the test sets decreased to varying degrees across regions. This train–test gap suggests that the random split may overestimate predictive performance, particularly in PRD, where the discrepancy between training and testing R^2^ was most pronounced. Test-set performance remained moderate in BTH and CDCQ but was weaker in MY, YRD, and especially PRD, indicating regional differences in model transferability even under the conventional validation setting.

**Table 2:** Training and test performance of the RF models across the five UAs.

UA	Samples	Train	Test	R2 (train)	RMSE (train)	MAE (train)	R2 (test)	RMSE (test)	MAE (test)
BTH	11,996	9,596	2400	0.9565	0.3805	0.2934	0.6924	0.9940	0.7611
CDCQ	4,961	3,968	993	0.9526	0.5876	0.4191	0.6965	1.5440	1.1139
MY	8,200	6,560	1640	0.9379	0.5301	0.4132	0.5627	1.4090	1.0961
YRD	18,457	14,765	3692	0.9361	0.4828	0.3670	0.5402	1.2971	0.9885
PRD	8,304	6,643	1661	0.9099	0.7789	0.6096	0.3337	2.1565	1.6957

To further evaluate model performance under spatially independent conditions, regular spatial block cross-validation was conducted (Table 3). Compared with the random train-test split, all datasets showed lower performance under spatial validation, indicating that spatial autocorrelation in the random split likely inflated model performance. Among the five regions, BTH and YRD showed relatively stronger spatial generalization, whereas PRD showed the weakest performance under spatial cross-validation. RMSE and MAE values were also consistently higher under spatial validation than under the random split. Taken together, these results suggest that the models are more reliable for identifying within-UA statistical association patterns than for strong spatial extrapolation to unseen urban subregions.

**Table 3:** Spatial block cross-validation performance of the RF models across the five UA datasets.

UA	Samples	Folds	R2 (mean)	RMSE (mean)	MAE (mean)	R2 (pooled)	RMSE (pooled)	MAE (pooled)
BTH	11,996	5	0.3220	1.3489	1.0514	0.4134	1.3924	1.0952
CDCQ	4,961	5	0.1483	2.2211	1.7336	0.2773	2.3141	1.7803
MY	8,200	5	0.3040	1.7977	1.4360	0.3290	1.7433	1.3793
YRD	18,457	5	0.3533	1.5381	1.1837	0.3668	1.5204	1.1732
PRD	8,304	5	0.1106	2.4156	1.9344	0.1443	2.4096	1.9178

### 3.3 Model-associated importance of variables associated for LST

Before interpreting variable importance, we note that several environmental and urban-form predictors are conceptually related and may contain overlapping information. Therefore, the relative contributions of individual predictors should be interpreted as model-associated importance within the multivariable framework rather than as fully independent effects.

**Figure 4:**
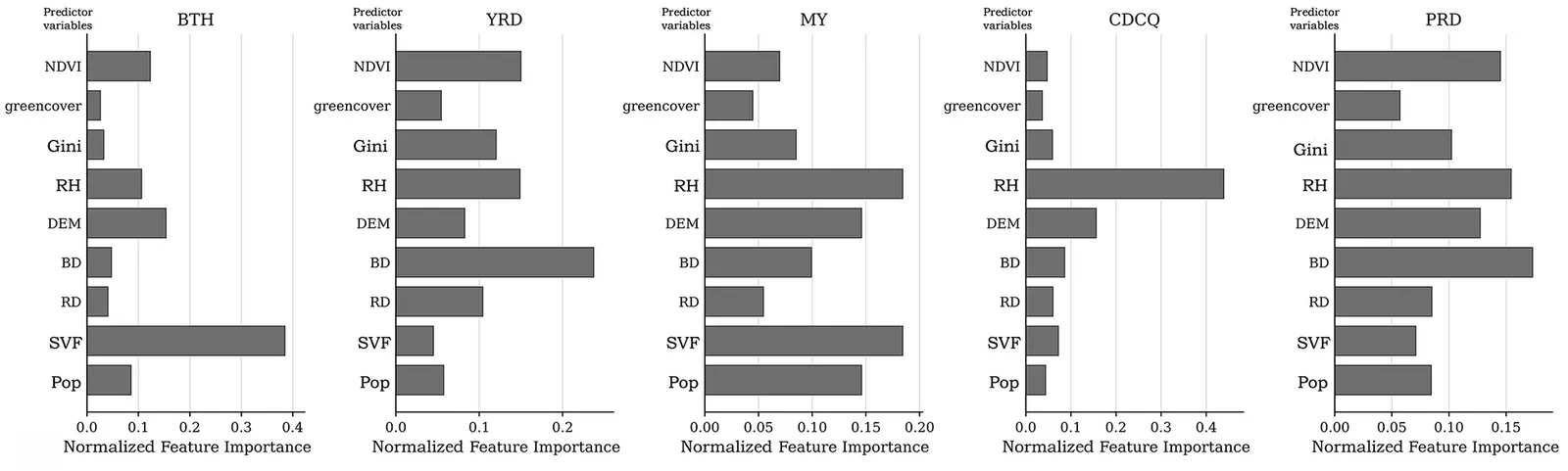
Random forest variable importance for LST across five UAs.

To identify and quantify the relative contributions of environmental and urban-form variables to LST, we extracted variable importance metrics after training the RF models and further report the random forest importance in each UA (Table 4). Based on these results, we report the importance weights of all variables in a fixed order consistent with the model inputs and present their relative contributions to model predictions (Figure 4). The numerical comparison indicates that green-related variables contribute consistently across the five major UAs, while their relative weights exhibit pronounced regional differences. Among the green-related variables, NDVI generally shows greater importance than green cover in most UAs, whereas the weighted Gini coefficient is more prominent in some regions.

**Table 4:** RF importance of predictors across the five UAs.

UA	BTH	YRD	MY	CDCQ	PRD
Variable	Importance				
NDVI	0.123	0.150	0.070	0.047	0.145
Green cover	0.026	0.055	0.045	0.037	0.057
Gini	0.033	0.120	0.085	0.059	0.102
RH	0.106	0.149	0.184	0.439	0.154
DEM	0.154	0.082	0.146	0.156	0.127
BD	0.048	0.237	0.099	0.086	0.173
RD	0.041	0.104	0.054	0.060	0.085
SVF	0.385	0.045	0.184	0.072	0.071
Pop	0.085	0.057	0.146	0.044	0.084

Across UAs, the contribution profiles of green-related variables were broadly similar in PRD and YRD. In both UAs, NDVI received a comparatively higher importance weight, green cover ranked second, and the weighted Gini coefficient, representing inequality in local green resource availability, exhibited a moderate relative importance. In MY, the relative importance of the weighted Gini coefficient was more pronounced. By contrast, the overall importance weights of green-related variables were lower in CDCQ and BTH. Other variables nevertheless provided important contextual constraints. For example, BD and SVF, two urban form variables, had relatively high importance weights in some UAs. The importance of other natural environmental variables, including RH and DEM, varied substantially across UAs. Socio-economic and urban built-environment variables, such as Pop and RD, showed weaker overall contributions, although they retained non-negligible importance in certain UAs.

### 3.4 Model-associated patterns of variables to LST

#### 3.4.1 Green-related variables

Using SHAP, we visualized the major variables associated with LST and their nonlinear dependence patterns. Overall, green-related variables, including NDVI, green cover and the weighted Gini coefficient, exhibited clear model-associated cooling contributions across all UAs. However, their dependence curves showed pronounced nonlinearity and apparent turning ranges. The Gini-related pattern further indicated that the spatial distribution of green resources is associated with the thermal environment in ways that differ substantially across UAs.

**Figure 5:**
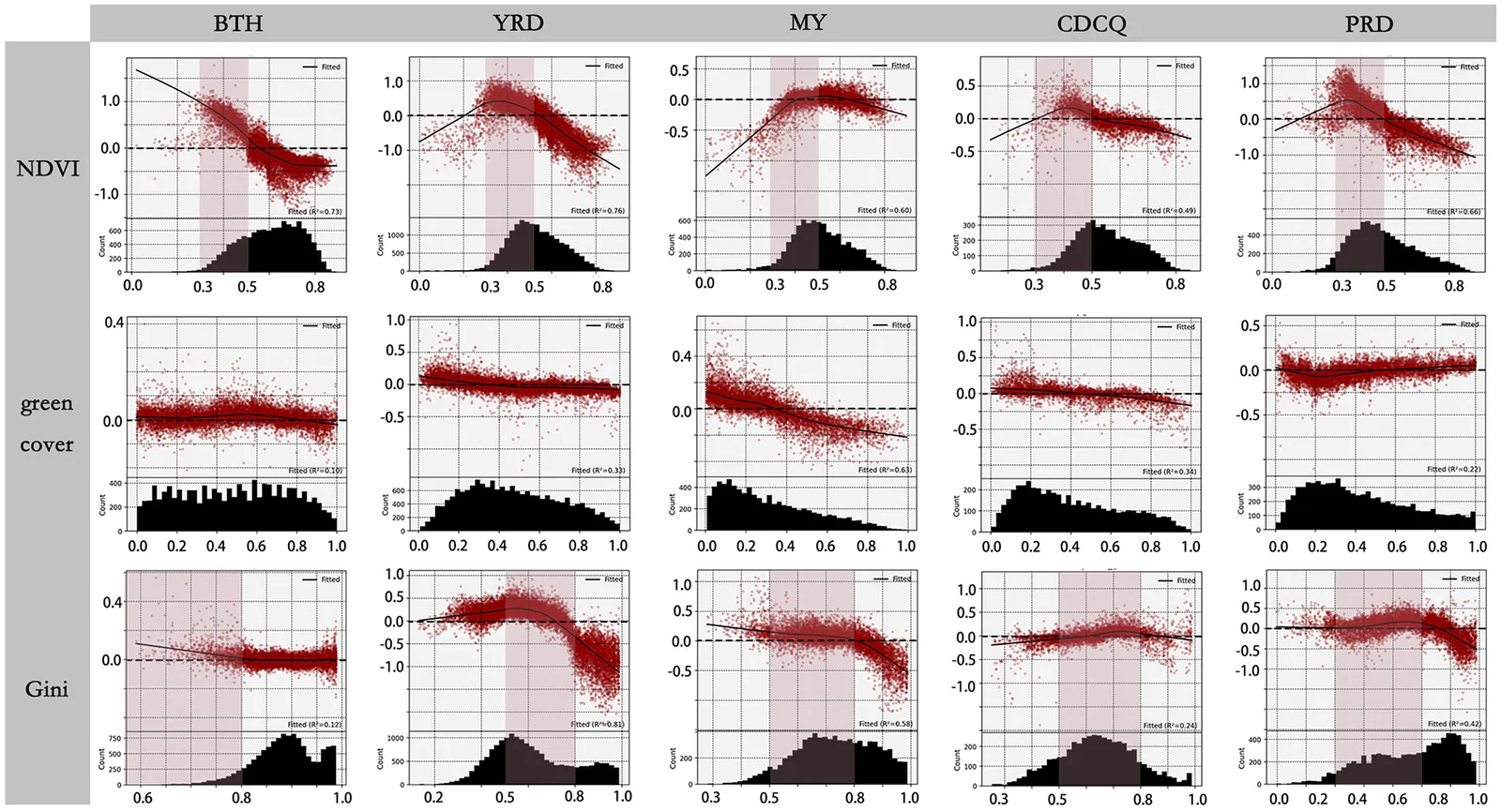
SHAP dependence plots of green-related variables for LST.

Figure 5 shows that the SHAP dependence curves of the NDVI in PRD, YRD, MY, and CDCQ generally exhibit an increase followed by a decrease. This pattern indicates that the dependence curve does not begin to decline rapidly until NDVI reaches a moderate level, suggesting an enhanced cooling-associated contribution beyond this range. Cross-UA comparisons suggest that the apparent turning range in the NDVI–LST relationship is broadly concentrated between 0.3 to 0.5. The main differences across UAs occur in the magnitude of the negative SHAP contributions after the curves begin to decline. In most UAs, higher NDVI values are associated with a stronger contribution to reducing model-predicted LST. Relative to the other UAs, the fitted curve for MY declines less steeply in the high NDVI range. In BTH, the fitted relationship is overall monotonically decreasing and tends to level off when NDVI exceeds 0.7, suggesting a possible saturation of the cooling-associated contribution at high NDVI levels.

Compared with NDVI, the SHAP dependence curves for the green cover variable in most UAs lie closer to the *y* = 0 reference line, indicating a relatively weaker marginal contribution to explaining LST. This suggests that increases in green cover alone do not necessarily associate with a pronounced negative SHAP contribution in the models. Nevertheless, inter-UA differences are evident. For example, the fitted curve for MY is monotonically decreasing.

For the weighted Gini coefficient, the dependence curves in PRD, YRD, and CDCQ all exhibit an increase followed by a decrease, with the apparent turning range broadly occurring between 0.5 and 0.8. This pattern indicates a nonlinear relationship with model-predicted LST. At low to moderate values, the SHAP contribution tends to increase. At higher values, the SHAP contribution shifts downward.

This pattern varies across UAs. YRD shows the most pronounced downward trend and the strongest marginal SHAP change, whereas CDCQ exhibits only a slight decline and its curve remains closer to the *y* = 0 reference line. In addition, the curve for BTH is overall monotonically decreasing and relatively gentle, and it approaches the *y* = 0 reference line more closely when values exceed 0.8. Similar to the NDVI dependence pattern in this UA, this suggests that the marginal model-associated relationship weakens or tends to level off under extremely high Gini values. MY also shows an overall monotonic decrease, but the decline accelerates within the 0.8–1.0 range, suggesting a stronger cooling-associated contribution under the high-value pattern captured by this variable.

#### 3.4.2 Other natural environmental variables

**Figure 6:**
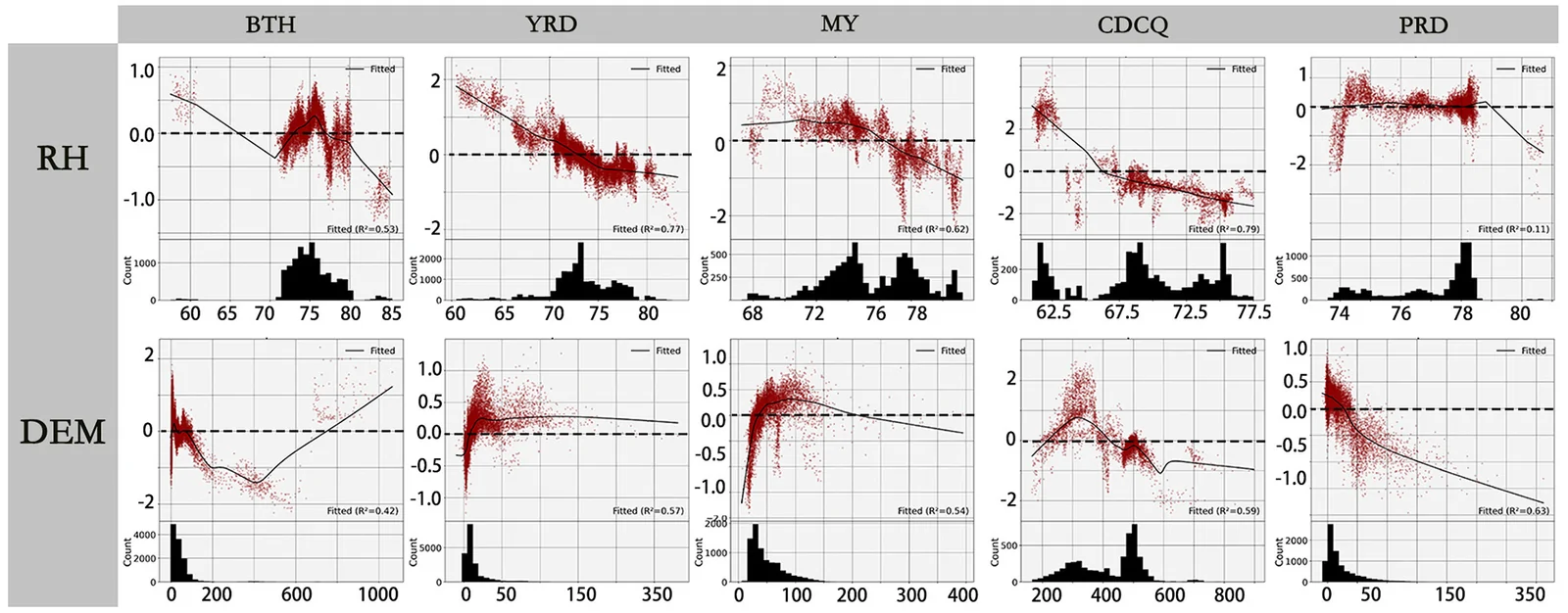
SHAP dependence plots of other natural environmental variables for LST.

Besides green-related variables, natural environmental variables also play a role in shaping LST across UAs, with RH and DEM showing non-negligible effects. Figure 6 shows that RH generally showed a stable negative model-associated relationship with LST. For RH, the results for YRD, BTH, MY, and CDCQ consistently show a clear negative association. The curves often flatten in the high-RH range, suggesting diminishing marginal cooling contributions. In more humid UAs, such as PRD and MY, the effective range tends to be broader, whereas in relatively drier or more seasonally variable regions, such as BTH, the relationship is more likely to show a nonlinear transition or leveling off.

DEM shows markedly higher dispersion of scatter points, and its dependence curves more readily display complex nonlinear patterns. This implies that elevation appears in the model primarily as a topographic background variable and its SHAP pattern may be associated with vertical temperature lapse rates, altitudinal differentiation of land surface types, and elevation-related gradients in urban development intensity. In terms of the overall direction, most UAs show a tendency for lower SHAP values at higher elevations. YRD, MY, and CDCQ exhibit an increase followed by a decrease to varying degrees. The increasing trend occurs within the 0–50m range for YRD and MY, and within the 0–300m range for CDCQ. In areas with pronounced relief, such as CDCQ, the segmentation and fluctuations in the DEM relationship are more evident, indicating stronger topographic heterogeneity in the model-associated pattern.

#### 3.4.3 Socio-economic and urban built-environment variables

**Figure 7:**
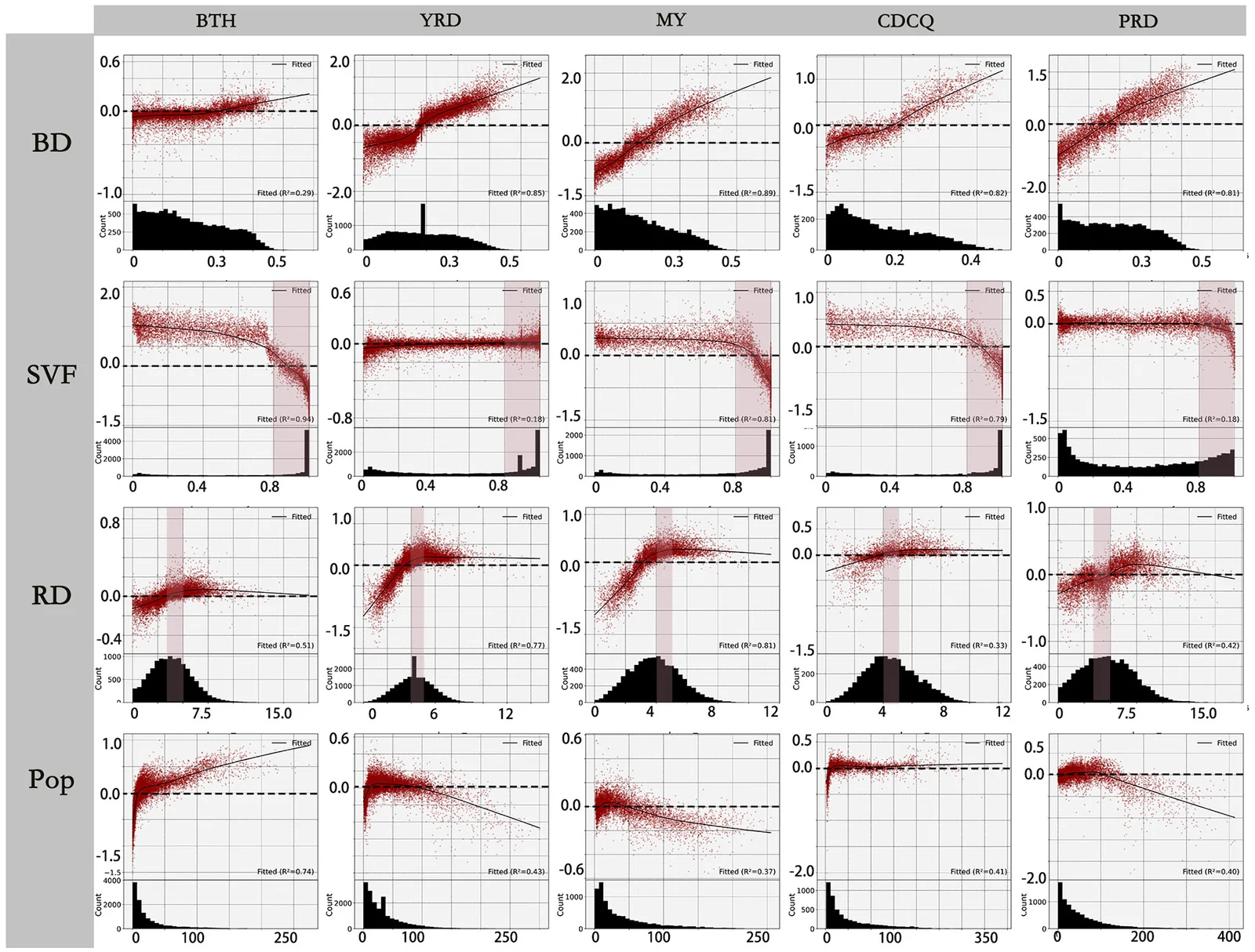
SHAP dependence plots of socio-economic and urban built-environment variables for LST.

Figure 7 shows that all UAs exhibit a monotonic increasing trend for BD, with an increasingly positive contribution to LST. The scatter points in PRD, YRD, and MY are more tightly clustered around the fitted line, and the curve shapes are clearer, suggesting that BD has stronger and more stable model-associated importance for LST prediction in these three UAs. For SVF, the responses in BTH, CDCQ, and MY are generally stable or slowly decreasing at low to intermediate values. When SVF enters the higher range, particularly between 0.8 and 1.0, the fitted curves decline more rapidly and shift toward a stronger negative contribution, implying that higher SVF is associated with lower model-predicted LST in this value range.

For RD, all UAs show a highly consistent nonlinear pattern. The fitted curves rise rapidly at low values, then flatten after RD reaches an apparent turning range of 4 to 5, with a slight downturn at higher values in some UAs. The dependence curve patterns for Pop are similar in PRD, MY, and YRD. As population density increases, SHAP values increase rapidly in the low range and then gradually shift to a flatter or decreasing trend, resulting in a weak nonlinear pattern characterized by an initial increase followed by a decrease. In BTH, apart from the increase at low values, the relationship is overall monotonically increasing, indicating a persistent strengthening of the warming contribution. In addition, the distribution histograms indicate that, across UAs, samples are heavily concentrated within the low value ranges where the curves increase most rapidly.

#### 3.4.4 Interaction patterns among NDVI, Gini, and BD

**Figure 8:**
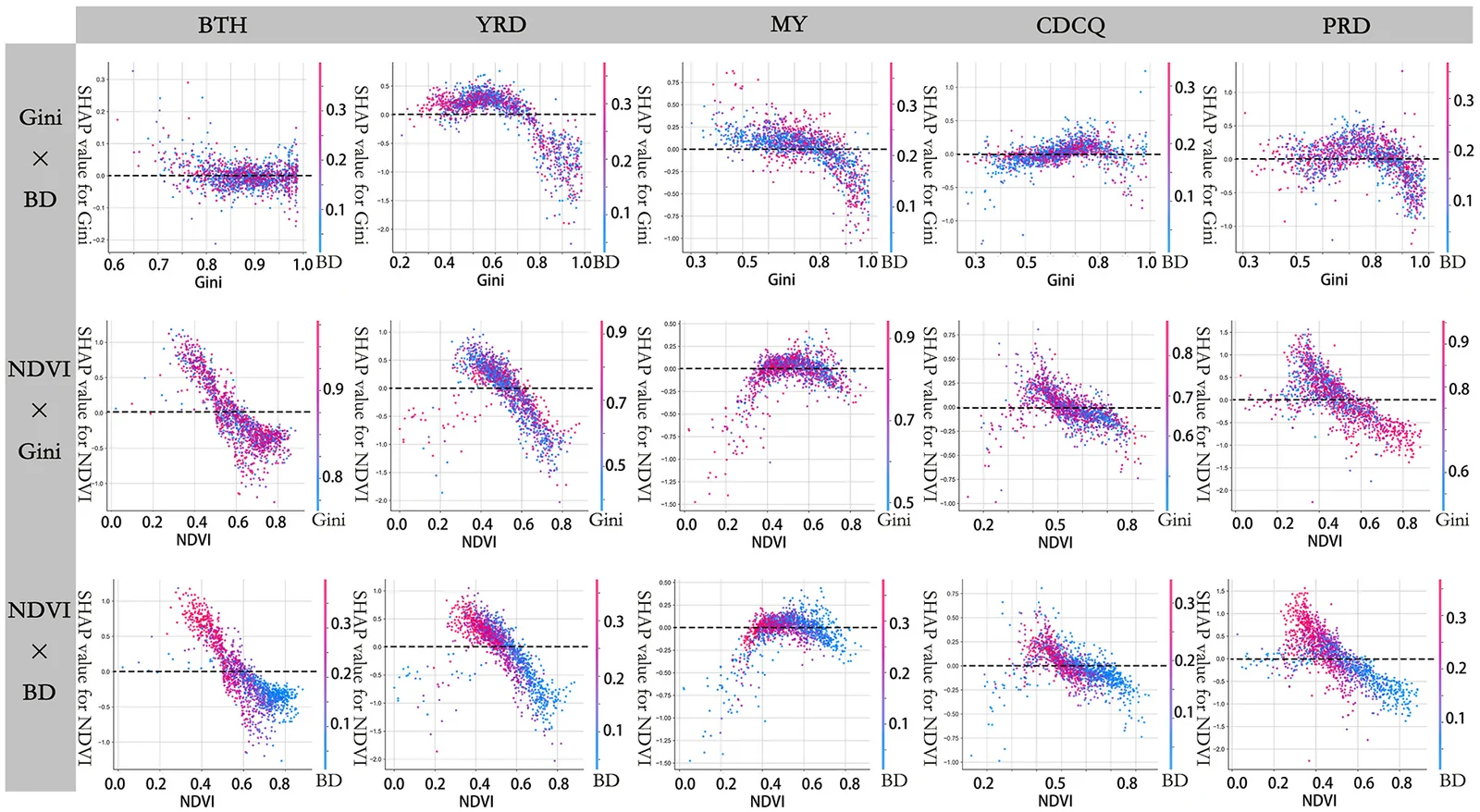
SHAP dependence plots showing interaction patterns among NDVI, Gini, and BD.

To further examine whether the model-associated effects of key green-related variables vary under different urban development intensities, we visualized SHAP dependence plots with interaction coloring for three variable pairs: Gini × BD, NDVI × Gini, and NDVI × BD (Figure 8). Overall, the plots show that the SHAP patterns of NDVI and the weighted Gini coefficient vary across levels of BD and across UAs.

For the Gini × BD interaction, positive SHAP contributions at moderate Gini values are more evident under higher BD in YRD, MY, and PRD. The downward shift at high Gini values is also clearer when BD is high. For the NDVI × Gini interaction, the negative SHAP contribution of NDVI becomes more apparent in the moderate-to-high NDVI range, although the magnitude of this decline differs across Gini levels and UAs. For the NDVI × BD interaction, the negative SHAP contribution of NDVI is generally weaker under higher BD at low-to-moderate NDVI levels, but becomes more evident after NDVI exceeds its turning range.

## 4 Discussion

### 4.1 Spatial differentiation of the weighted Gini coefficient of green resource availability across built-up areas in different UAs

Spatially, the **weighted Gini coefficient** is characterized by distinct pockets of elevated values, whereas lower values are more diffusely distributed and, in some areas, align into elongated corridors or broader low-value zones. In conjunction with Figure 3, high value clusters suggest a stronger local concentration of green resources within the surrounding neighborhood, whereas low-value corridors are more compatible with relatively even local distributions. In map-based terms, low value corridors may coincide with continuous green corridors, waterfront green belts, clustered park systems, or relatively balanced neighborhood scale green cover networks. Notably, CDCQ—the westernmost of the five UAs—shows the lowest mean weighted Gini coefficient, broadly consistent with Wu and Kim (49), who found that cities in western China tend to exhibit more equitable access to green space than those in southern China, along the southern coast, and in the northeast.

At the UA scale, some regions show an inner-to-outer gradient, with peripheral areas more likely to exhibit relatively high weighted Gini values. This spatial pattern may be associated with uneven green resource distribution between urban cores and peripheral built-up areas. One possible explanation, supported by previous studies, is that industrial suburbanization and land-use expansion in urban fringes may coincide with reduced green-space continuity or availability in some locations (71, 72). Evidence from Shanghai also shows a spatial overlap between industrial clusters along the outer ring and areas with poor green resource availability (73). In addition, low Gini values appear more concentrated in some core cities, which may be compatible with a relatively better population–service match in larger urban systems (39, 74).

### 4.2 Heterogeneity of green-related variables based on SHAP interpretation

Greenness level and green cover area are both important variables for evaluating the role of green space in environmental justice and public health (75). In the present models, NDVI showed higher importance than green cover in most UAs. This pattern may reflect that NDVI captures variation in vegetation condition more effectively than green cover proportion alone, whereas green cover may contain less information about vegetation vigor and may also overlap with spatial configuration effects (76).

Across the UAs, green-related variables exhibited distinct regional contrasts in importance weights. PRD and YRD showed broadly consistent profiles, with NDVI ranked highest, green cover ranked second, and the weighted Gini coefficient contributing at a moderate level. In MY, the weighted Gini coefficient was comparatively more influential, whereas CDCQ and BTH displayed generally lower overall weights for green-related variables. Overall, these differences suggest that the relative model importance of greenness and inequality-related indicators is not uniform across UAs.

### 4.3 Heterogeneity in the model-associated patterns linking green-related variables and LST across UAs

The weighted Gini coefficient of local green resource availability can display an increase followed by a decrease, or a stronger cooling-associated contribution at the high value end, which may correspond to two processes. The first process occurs when the weighted Gini coefficient rises from low to moderate levels. In this range, green resources become more unevenly distributed across local space, while some areas remain relatively underserved. These areas may also coincide with more intensive built-up conditions. Under this configuration, the model identifies a more positive association with LST as green resource inequality increases.

The second process may occur after the weighted Gini coefficient exceed a turning range. In this range, green resources may become more spatially concentrated, potentially reflecting the presence of a limited number of large and relatively continuous green patches or corridors. Under such a configuration, SHAP shows a stronger negative association at higher green resource inequality levels.

The relationship between the weighted Gini coefficient and LST is not simply monotonic. This distribution-based indicator does not directly represent a better or worse thermal outcome (77). Rather, it can be interpreted as a proxy for the spatial structure of local green resource provision, including the degree of concentration, continuity, or fragmentation. Because its meaning depends on the broader supply context, a lower Gini coefficient in UAs with relatively abundant green resources may indicate more universal provision, whereas in UAs with insufficient overall supply, it may instead reflect a more even distribution of limited green resources (54).

Differences in the post-turning slope provide a basis for cross-UA comparison under similar Gini coefficient values. YRD shows the most pronounced decline, indicating a stronger negative model-associated relationship in the higher value range. By contrast, CDCQ exhibits only a very small decline after the turning point, and its curve remains close to the zero line; BTH shows a similar pattern. In the extremely high inequality range above 0.8, further increases in the weighted Gini coefficient do not necessarily imply stronger green resource benefits. Instead, they may reflect further concentration or redistribution under the existing spatial configuration. In such cases, the marginal SHAP response may weaken or approach saturation (78).

Compared with the weighted Gini coefficient, the NDVI pattern is less complex. At low NDVI values, marginal increases in NDVI are not associated with an immediate negative SHAP response; instead, the estimated contribution becomes more positive. As NDVI rises into a moderate range, the dependence curve turns downward sharply and crosses into negative values, indicating a stronger cooling-associated contribution. In built-up areas, samples in the low NDVI range may correspond to urban expansion zones or transitional spaces with relatively intensive impervious surfaces (79). In such settings, limited vegetation may be insufficient to offset the thermal influence of the surrounding built environment. Accordingly, the model shows a more positive SHAP contribution within this interval. This pattern is broadly consistent with Zhang et al. (80). When NDVI exceeds approximately 0.3–0.5, the curves generally shift into a rapid decline. This suggests that the cooling-associated contribution of vegetation becomes more apparent beyond this range. The relative consistency of this turning range across multiple UAs indicates a potentially common nonlinear transition. This pattern is statistically compatible with the possibility that, beyond this NDVI range, vegetation-related cooling processes, such as shading and evapotranspiration may become more evident (81).

Differences in post-turning slopes across UAs indicate that the same increment in NDVI is not associated with equivalent marginal responses across regions. The smaller decline in MY at high NDVI values may reflect stronger background constraints under local hydrothermal conditions. By comparison, BTH shows a monotonic decline with a tendency to level off at high values, suggesting possible saturation of the marginal cooling-associated contribution. This pattern may be consistent with stronger climatic or environmental constraints in this region. After a certain greenness level is reached, vegetation cooling can be constrained by low soil moisture. In northern China, afforestation initiatives have often emphasized farmland protection, soil conservation, and desertification control (82).

The interaction plots further indicate that the model-associated relationships of NDVI and the weighted Gini coefficient with LST are context-dependent. Specifically, the SHAP patterns of these green-related variables vary with BD and the level of green resource inequality. This suggests that their nonlinear associations should be interpreted together with urban development intensity and local spatial distribution structure rather than as isolated effects.

### 4.4 Heterogeneity in model-associated patterns of other variables across UAs

The dependence curves indicate that the associations between built-environment variables and LST are not uniform across UAs. Among them, BD shows a persistently increasing SHAP pattern across all UAs, suggesting a stable positive association with model-predicted LST. This pattern is consistent with previous studies reporting higher thermal loads in more intensively built environments (83, 84). Notably, the scatter points are more concentrated, and the curves are clearer in the plain dominated UAs of PRD, YRD, and MY.

For SVF, BTH, CDCQ, and MY show relatively weak changes at low-to-intermediate values, followed by a stronger negative SHAP shift when SVF reaches the high range of approximately 0.8–1.0. This pattern suggests that higher openness is associated with lower model-predicted LST mainly in the upper value range. Such a pattern is broadly compatible with previous studies linking urban openness to improved thermal conditions (85).

For Pop, the low-to-moderate range is generally associated with a rising SHAP pattern in several UAs, which is consistent with the positive statistical association between Pop and LST reported by Parvez and Aina (86).

Overall, the SVF variable and the building density variable show relatively high importance weights in some UAs, suggesting that urban morphological context plays a stronger role in the corresponding models. This result is consistent with Badaro-Saliba et al. (87). By contrast, DEM does not emerge as a key variable within this set, which differs from the within neighborhood vertical analysis reported by Chen et al. (89). The importance of RH and DEM varies more across regions, implying that background environmental constraints differ among UAs. These cross-regional differences should be interpreted as heterogeneity in model-associated patterns.

### 4.5 Planning and management implications

In response to urban climate risks, the present results suggest several threshold-informed implications for planning and management. First, greening strategies should not focus solely on expanding area, but also on improving vegetation condition. Across most UAs, the NDVI dependence curves begin to shift toward clearer negative SHAP contributions when NDVI reaches an approximate turning range of 0.3–0.5. This suggests that fragmented or low-quality vegetation below this range may not generate equally strong cooling-associated contributions, whereas moving urban greening into at least a moderate NDVI range may be more effective. Second, the results highlight that the thermal relevance of green resource distribution is nonlinear. In several UAs, the weighted Gini coefficient shows a turning range around 0.5–0.8, after which the SHAP pattern shifts downward. This implies that planning should distinguish between simply equalizing low levels of green provision and improving the spatial structure of green resources. In practice, this means combining more even neighborhood-level service coverage with the preservation of larger or more continuous green patches where they provide strong model-associated cooling benefits.

### 4.6 Limitations

This study has several limitations that should be addressed in future research. First, we used the weighted Gini coefficient of green resource availability to characterize spatial inequality. However, this variable primarily reflects the degree of dispersion of accessible resources across spatial units, and it is not directly equivalent to the green cover benefits that residents actually receive. Future studies should further differentiate and incorporate, in parallel, variables capturing area based equity, quality based equity, and ecosystem service inequality (74) to reduce interpretive ambiguity. Second, LST is a proxy for the thermal environment, and it does not fully represent human heat exposure. This study used LST to capture thermal responses, but LST is not equivalent to near surface air temperature, thermal comfort, or residents' realized heat exposure. Future work should couple LST with population distribution, activity intensity, and the exposure of vulnerable groups to conduct heat exposure assessments (88), and incorporating street scale information would further strengthen the direct policy relevance for health equity. Finally, our interpretation of the relationships between LST and multiple factors, including equity related variables, is based on nonlinear effect statistical associations derived from the model, rather than on direct causal relationships. The underlying mechanisms should be further identified and examined in future research. In addition, although we introduced spatial block cross-validation to provide a stricter validation setting, model performance declined under spatially independent evaluation. This suggests that the present framework is more suitable for identifying within-UA statistical association patterns than for strong extrapolation to unseen urban subregions or to other cities without recalibration.

## 5 Conclusion

Using a Random Forest model and a SHAP based interpretability framework, this study systematically examined nonlinear relationships between multidimensional urban variables and LST across five UAs. The analysis jointly considered vegetation and other green-related variables, green resource availability inequality, natural background variables, built-environment and socio-economic variables. The results show that green-related variables make consistent contributions across the five UAs, but their contribution structures differ markedly. Compared with green cover as a quantity-oriented measure, NDVI shows stronger explanatory power in most UAs. This suggests that urban cooling is more strongly associated with vegetation vigor and quality than with simple increases in green area. In some UAs, the cooling-associated contribution of NDVI levels off at high values, suggesting possible constraints from hydrothermal background conditions and urban form. By contrast, the marginal contribution of green cover in most UAs is closer to that of background variables, implying that increasing green cover proportion alone does not necessarily produce equivalent cooling, as its effectiveness is moderated by spatial configuration, surrounding impervious intensity, and climatic conditions. The relationship between the weighted Gini coefficient of green resource availability and LST is also strongly nonlinear and varies across UAs, indicating that this Gini-based distributional indicator reflects not only distributional balance but also the structural organization of green space service provision. In cross-regional comparisons, YRD is more sensitive to equity changes and shows stronger marginal effects, whereas CDCQ exhibits weaker marginal effects, possibly due to attenuation by complex terrain and surface heterogeneity. Beyond green-related factors, urban morphology also shows clear associations with LST. BD shows a stable warming-associated contribution across all UAs, indicating the importance of high-intensity impervious development. Overall, by adopting a multi-UA perspective and leveraging SHAP to enhance model interpretability, this study clarifies how green cover, equity, and urban form are associated with thermal environments across typical Chinese UAs. It also advances the analysis from prediction to the interpretation of nonlinear and process-related patterns. These findings provide actionable quantitative evidence for urban planning and heat-risk governance. Green space strategies should move beyond simple area expansion to prioritize vegetation quality, spatial continuity, and UA-specific threshold intervals under different climatic backgrounds. Meanwhile, optimization of green resource distribution should be tailored to local conditions, especially by improving green space configuration and service coverage in regions that are more sensitive to Gini-related change.

## Data Availability

The original contributions presented in the study are included in the article/supplementary material, further inquiries can be directed to the corresponding author.
